# A novel Zn chelate (TSOL) that moves systemically in citrus plants inhibits growth and biofilm formation of bacterial pathogens

**DOI:** 10.1371/journal.pone.0218900

**Published:** 2019-06-24

**Authors:** Hajeewaka C. Mendis, Ali Ozcan, Swadeshmukul Santra, Leonardo De La Fuente

**Affiliations:** 1 Department of Entomology and Plant Pathology, Auburn University, Auburn, AL, United States of America; 2 NanoScience Technology Center, University of Central Florida, Orlando, FL, United States of America; 3 Department of Chemistry, University of Central Florida, Orlando, FL, United States of America; 4 Department of Materials Science and Engineering, University of Central Florida, Orlando, FL, United States of America; 5 Burnett School of Biomedical Sciences, University of Central Florida, Orlando, FL, United States of America; LSU Health Sciences Center School of Dentistry, UNITED STATES

## Abstract

Ternary solution (TSOL) is a novel Zn chelate-based systemic antimicrobial formulation designed for treating citrus bacterial pathogens ‘*Candidatus* Liberibacter asiaticus’ and *Xanthomonas citri* subsp. *citri*. TSOL is a component of MS3T, a novel multifunctional surface/sub-surface/systemic therapeutic formulation. Antimicrobial activity of TSOL was compared with the antimicrobial compound ZnO against *X*. *citri* subsp. *citri* and ‘*Ca*. L. asiaticus’ surrogate *Liberibacter crescens* in batch cultures. *X*. *citri* subsp. *citri* and *L*. *crescens* were also introduced into microfluidic chambers, and the inhibitory action of TSOL against biofilm formation was evaluated. The minimum inhibitory concentration of TSOL for both *X*. *citri* subsp. *citri* and *L*. *crescens* was 40ppm. TSOL was bactericidal to *X*. *citri* subsp. *citri* and *L*. *crescens* above 150 ppm and 200 ppm, respectively. On the contrary, ZnO was more effective as a bactericidal agent against *L*. *crescens* than *X*. *citri* subsp. *citri*. TSOL was more effective in controlling growth and biofilm formation of *X*. *citri* subsp. *citri* in batch cultures compared to ZnO. Time-lapse video imaging microscopy showed that biofilm formation of *X*. *citri* subsp. *citri* was inhibited in microfluidic chambers treated with 60 ppm TSOL. TSOL also inhibited further growth of already formed *X*. *citri* subsp. *citri* and *L*. *crescens* biofilms in microfluidic chambers. Leaf spraying of TSOL showed higher plant uptake and systemic movement in citrus (*Citrus reshni*) plants compared to that of ZnO, suggesting that TSOL is a promising antimicrobial compound to control vascular plant pathogens such as ‘*Ca*. L. asiaticus’.

## Introduction

Huanglongbing (HLB) is the most devastating disease of citrus worldwide [1], and it is associated with Gram-negative, phloem-restricted and non-cultured bacteria ‘*Candidatus* Liberibacter asiaticus’ (*C*Las), ‘*Candidatus* Liberibacter africanus’ (*C*Laf) and ‘*Candidatus* Liberibacter americanus’ (*C*Lam) [2]. HLB can be transmitted from tree to tree by grafting and through citrus psyllid vectors *Trioza erytreae* in Africa and Asian citrus psyllid (ACP) *Diaphorina citri* in Asia and the Americas [2]. HLB associated with *C*Las is found in Asian countries, as well as in Brazil and USA [2]. HLB symptoms include yellowing of shoots, stunting and declining of trees, deformed (lop-sided) and poorly colored (greening) fruits, aborted seeds in fruits and weak root systems with relatively few fibrous roots [3]. Progression of symptoms can be very fast and severe symptoms have been observed within one to five years after symptoms appear, depending on the age of the tree at the time of infection and the number of infections per tree. Yield reduction and a decrease in fruit quality occur with the increase in disease severity [1, 4–7]. Yield reduction in HLB infected trees occur at a faster rate compared to the development of symptoms [3, 8].

There are three management practices recommended for HLB. These include use of insecticides to control ACP to minimize the spread of inoculum, removal of HLB-infected trees to reduce the sources of inoculum, and planting HLB-free trees to prevent introduction of new inoculum [9, 10]. Insecticides such as Imidacloprid, Fenpropathrin, Chlorpyrifos, Thiamethoxam and Dimethoate are considered effective against ACP [9, 11]. Effective control of ACP using insecticides largely depends on environmental conditions, ACP control in the surrounding areas and removal of diseased trees to reduce inoculum loads [9]. Therefore, control of HLB through insecticides by controlling ACP is difficult in areas endemic to HLB [9]. A study in Brazil has shown that there was no relationship between the number of insecticide sprays and HLB incidence; and systemic insecticide applications in localized areas were not efficient in preventing *C*Las transmission by infectious psyllids from external sources [12]. Another study done by Hall et al. (2013) concluded that none of the ACP management programs they investigated prevented introduction and spread of HLB [9]. Berhamin-Filho et al. (2009) reported that eradication of symptomatic trees is more effective in controlling HLB incidence than use of insecticides [9, 12]. However, eradication of symptomatic trees may not be a viable solution to control HLB, since *C*Las has a long and highly variable incubation time. Approximately 1 to 2.5 years of incubation time is estimated for 7 to 10 years old trees, whereas it could be only 6 to 12 months for younger plants. Therefore, *C*Las-infected but asymptomatic trees may act as a source of infection [1]. Within an infected plant, bacterial populations vary in different parts of the tree and may be below the threshold of PCR detection limit, but grafting experiments still can result in new infected trees [1, 13–15]. PCR detection in the field is generally negative or inconclusive for 9 to 12 months, or longer, after trees become infected [16]. One of the solutions to HLB is to develop resistant cultivars through breeding programs. However, it would require many years of field testing to determine horticultural suitability of a new cultivar [16]. Even the most promising resistant cultivars could become susceptible as a result of the pathogen acquiring resistance through mutations [16]. There are reports of new varieties with enhanced resistance to HLB through the introduction of resistance genes [17, 18]. Consumer acceptance of such technologies could play an important role in commercialization of the end product.

*Xanthomonas citri* subsp. *citri* (Xcc) is another important pathogen that causes citrus canker in Florida [19]. It is a serious disease in commercial citrus cultivars such as grapefruit (*Citrus paradisi* Macf.) and sweet orange (*Citrus sinensis* (L.) Osbeck) [20]. Xcc is an epiphyte that infects host plants through stomata and wounds [19]. Raised corky lesions on twigs and fruit, defoliation, twig dieback, premature fruit drop and general decline symptoms are associated with citrus canker [19, 20]. Control of citrus canker in susceptible citrus cultivars is difficult when the plants are grown in tropical and subtropical areas [21, 22]. Xcc can infect new host plants from lesions on infected trees when there is moisture on the lesion surface [23]. Rain water splashes can disperse Xcc at short ranges whereas windblown rain droplets can disseminate Xcc at medium to long ranges [19]. Copper bactericides have been successful in controlling citrus canker but there are several disadvantages to using copper fungicides, including development of copper resistance in Xcc populations and phytotoxicity in citrus soils as a result of accumulation of copper [24–26]. Therefore, Zn-based bactericides could provide a better alternative to copper-based bactericides in the future [20].

In this study, we evaluated the antimicrobial properties of a novel Zn-chelate, ternary solution (TSOL), compared to the known antimicrobial compound ZnO. TSOL has been designed as a systemic antimicrobial compound [27] and is one of the main active ingredients in the experimental formulation MS3T (Multifunctional, surface/subsurface/systemic therapeutic) [27]. Zn-chelates have been used as a fertilizer in agriculture [28] and foliar application of Zn-chelates has been found to be more effective than application of ZnSO_4_ to alleviate Zn deficiency in cereals [29]. Antimicrobial properties of many Zn-chelates have been reported previously [30]. Wang et al. (2004) found that Zn-chelates have 4–16 times lower MIC compared to that of ZnSO_4_. The effectiveness of Zn-chelates was consistent with both Gram-positive and Gram-negative bacteria [30]. ZnO, the most common form of Zn that has been used as an antimicrobial compound, has antimicrobial activity against Gram-positive bacteria such as *Bacillus subtilis* and *Staphylococcus aureus* [31–33] and Gram-negative bacteria such as *Pseudomonas aeruginosa*, *Campylobacter jejuni* and *Escherichia coli* [34–38]. Release of reactive oxygen species and Zn-ions are responsible for the antimicrobial properties of ZnO [39, 40]. However, bulk ZnO may have limited uptake and systemic movement in plants as a result of its particle size and is not effective against systemic plant pathogens such as *C*Las.

The objective of this work was to evaluate effectiveness of the Zn-chelate TSOL to control growth and biofilm formation of *Liberibacter crescens* (Lcr) and Xcc in vitro in batch cultures and under constant nutrient flow conditions in microfluidic chambers (MC) mimicking the plant vascular system. Since *C*Las could not be maintained in culture, we used Lcr as a surrogate for *C*Las since it is the only member of the genus *Liberibacter* that has been cultured [41, 42]. We demonstrate that TSOL inhibits growth and biofilm formation of Xcc and Lcr in batch cultures and under flow conditions in MC. Furthermore, TSOL moves systemically in plants and can reach concentrations above MIC of Xcc and Lcr in leaves, stems and roots.

## Materials and methods

### Bacterial strains and growth conditions

*Xanthomonas citri* subsp. *citri* (Xcc) strain 306 was grown at 28°C with shaking at 150 rpm in Silva Buddenhagen (SB) medium or on SB agar plates [43]. *Liberibacter crescens* BT-1 (Lcr) [41] was grown at 28°C with shaking at 150 rpm in BM7 medium or on BM7 agar plates [41]. Stocks of the strains in 20% glycerol were kept frozen at -80°C.

### Synthesis and major properties of TSOL

TSOL is a Zn-based antimicrobial ternary complex consisting of zinc metal, salicylic acid, and hydrogen peroxide components synthesized according to Santra et al. [27]. A manuscript describing the formulation and molecular aspects of TSOL has been recently published by our group [44]. Salicylic acid can chelate metals with 2+ oxidation states and form a stable complex primarily through carboxyl and alcohol functional groups [45]. The stock solution of TSOL retains 4.4% (wt/wt) metallic Zn making it suitable for industrial-scale applications. As synthesized, TSOL solution was observed to be a white colloidal solution having a specific gravity of 1.14 g/cm^3^. The pH of TSOL stock solution was 3.4 and water (pH 6.5)-diluted TSOL solutions at 800 μg /mL and 1,600 μg /mL increased the pH to 5.0 and 4.8, respectively.

### Evaluation of growth inhibition and minimum inhibitory concentration (MIC) of Xcc and Lcr treated with TSOL and ZnO

Xcc cells were obtained from frozen stocks and re-streaked once on SB agar (SBA) plates incubated at 28°C before use. Liquid cultures of Xcc were started by collecting cells from a re-streaked SBA plate using 10 ml of SB medium. Xcc was inoculated to a final concentration of optical density (OD_600_) of 0.05 (approximately 5x10^5^ cells) in 2 ml of SB medium containing a TSOL or ZnO concentration gradient ranging from 0–150 ppm in 24-well tissue culture plates (VWR International, LLC). Two ml of uninoculated SB medium containing a TSOL or ZnO concentration gradient ranging from 0–150 ppm metallic Zn were used as blanks. The 24-well plates were sealed with Parafilm (Bemis Company, Inc) and surgical tapes (Durapore^TM^, 3M Healthcare) to prevent evaporation and incubated at 28°C with shaking at 150 rpm for 3 days. Total growth of Xcc was measured by OD_600_ using a plate reader (CYTATION 3 imaging reader, BioTek Instruments, Inc.). Planktonic growth was determined by transferring SB medium with TSOL or ZnO from inoculated and non-inoculated plates into new 24-well plates and measuring OD_600_. Biofilm growth was measured following the protocol described by O’Toole and Kolter (1998) using the original plate after removing liquid media for planktonic growth [46]. MIC was determined as described by Wang et al. [47].

A BM7 plate was inoculated with Lcr from a frozen stock culture and incubated at 28°C for 7–10 days. A liquid culture of Lcr was obtained by pouring 10 ml of BM7 medium into a 7-10-day old plate of Lcr. Lcr was inoculated to a final concentration of OD_600_ of 0.1 (approximately 5x10^5^ cells) and total, planktonic and biofilm growth were measured as described above following TSOL or ZnO treatment. Lcr batch cultures were incubated at 28°C with shaking at 150 rpm for 7 days.

### Determination of viability inhibition using alamarBlue assay

Xcc and Lcr were grown in 24-well plates in SB or BM7 medium, respectively, with a TSOL or ZnO concentration gradient ranging from 0–150 ppm as described above for 3 days. Two ml of uninoculated SB medium containing a TSOL or ZnO concentration gradient ranging from 0–150 ppm metallic Zn were used as blanks. An aliquot of 200 μl alamarBlue reagent (Serotec Inc, MS) was added to each well and incubated for 18 hours at 28^o^ C with shaking at 150 rpm. Absorbances at 570 nm and 600 nm were measured using the Cytation 3 plate reader. Xcc viability was assessed as the Percentage Difference in Reduction of alamarBlue (PDRAB), as described by the manufacturer. Briefly, the equation PDRAB = [(O2xA1)–(O1xA2)/ (O2xP1)–(O1xP2)]*100 was used, where O1 = molar extinction coefficient (E) of oxidized alamarBlue at 570 nm; O2 = E of oxidized alamarBlue at 600 nm; P1 = absorbance of positive growth control at 570 nm; P2 = absorbance of positive growth control at 600 nm; A1 = absorbance of test wells at 570 nm; A2 = absorbance of test wells at 600 nm. Percentage of viability inhibition, used for graphs, was calculated by this equation: Viability inhibition (%) = 100 –PDRAB.

### Determination of culturability by plating

Xcc and Lcr were grown in 24-well plates as described in the alamarBlue assay. An aliquot of 100 μl was taken from each well and plated onto SB agar or BM7 agar plates, respectively. SB plates with Xcc were incubated at 28°C for 6 days, and BM7 agar plates with Lcr were incubated at 28°C for 4 weeks.

### Evaluation of biofilm formation in microfluidic chambers (MC)

The fabrication of microfluidic chambers (MC) was performed as previously described by De La Fuente et al. (2007) [48]. A two parallel-channel MC design was used for all the MC experiments. The MC were 80 μm wide, 3.7 cm long and 50 μm deep. Each channel in the MC has two separate inlets to introduce bacterial growth media and bacteria and one outlet to collect flowing bacteria growth media. Inlets and outlets in MC were connected to tubing, syringes and syringe pumps (Pico Plus; Harvard Apparatus, Holliston, MA, USA) as described in De La Fuente et al. (2007). In separate experiments, either Xcc or Lcr bacterial suspensions obtained from SB or BM7 plates, respectively, were introduced to both MC channels through one of the two inlets and the media flow in the MC was set to 0.05 μl/min. Syringe pumps connected to bacterial inlets were stopped after 2–3 hours when bacteria were attached to the MC walls. Attached bacteria was allowed to form biofilm in the MC for 3 days (Xcc) or 7 days (Lcr) or until the bacteria attach to MC and form abundant biofilm in both channels. Lcr forms abundant biofilm in a modified BM7 medium [(with addition of 0.75 g per L of methyl-β-cyclodextrin and without fetal bovine serum (FBS)] named bBM7+0.75mβc [49]. Therefore, this medium was used for MC experiments with Lcr. A growth medium containing 60 ppm TSOL was introduced in one channel after biofilm formed in both channels, and changes in biofilm formation before and after TSOL treatment compared to biofilm from the untreated channel was observed with a Nikon Eclipse Ti inverted microscope (Nikon, Melville, NY) with phase-contrast and Normarski differential interference contrast (DIC) optics. Time-lapse images were recorded every 1 min (Xcc) or 2 min (Lcr) with a Nikon DS-Q1 digital camera (Diagnostic Instruments, Sterling Heights, MI, USA) connected to the microscope and controlled by NIS-Elements imaging software version 3.0 [48].

### Evaluation of TSOL uptake by citrus plants

A plant uptake study was performed using *Citrus reshni* (Cleopatra mandarin) seedlings in growth chambers (Panasonic Environmental Test Chamber, Model MLR-352H, Kadoma, Japan). Temperature and relative humidity were maintained at 35°C and 85%. Eleven-month old seedlings were foliar-sprayed using a hand-pump garden sprayer with the following treatments: 300 ml of TSOL, ZnO (ZnO grade 400, Zinc Oxide LLC) either at 800 μg Zn/mL or 1600 μg Zn/mL concentrations, and deionized water as a control. Foliar-spray concentrations were selected based on the field application rate of MS3T to control citrus canker. Leaf spraying was carried out in the morning to promote plant uptake through stomata and the pots were covered with Parafilm to prevent treatment solutions coming into contact with soil and seedling roots. Seedlings were returned to the growth chamber for two days prior to the analysis of Zn content. Plants were carefully removed from the soil and surface-bound material was removed as follows: whole plants were dipped in 40 ml of 1% detergent (Alconox, Alconox Inc.) in 2 L and gently rinsed for 10 minutes, and the rinsed twice with 2 L of DI water right after for 2 minutes. Afterwards, plants were rinsed in 2 L of 0.1% HCL _(aq)_ for 30 seconds and 2 L of DI water for 2 minutes in order to remove surface deposited mineral elements including Zn [50]. Leaves, roots and stem sections were separated after washing and left in a drying oven (Cabela’s Inc, Sydney, NE) at 45°C for 48h. The dried leaves, stems and roots were pulverized separately using mortars and pestles prior to acid digestion. One gram of dry powder of leaves and stems, and 0.5 grams of roots were acid digested following EPA method 3050B “Acid Digestion of Sediments, Sludge, and Soils” [51]. Zn concentrations in leaves, stems and roots were quantified using Atomic Absorption Spectroscopy (AAS).

### Statistical analysis

Treatment effects were analyzed by Kruskal-Wallis One Way Analysis of Variance on Ranks and significant differences between treatments and the non-treated control were analyzed using Dunn’s Method (tested at *P* < 0.05) using the SigmaPlot Software, Version 13.0 (Systat Software, Inc., San Jose California USA).

## Results

### Inhibition of growth and biofilm formation by TSOL

**Fig 1:**
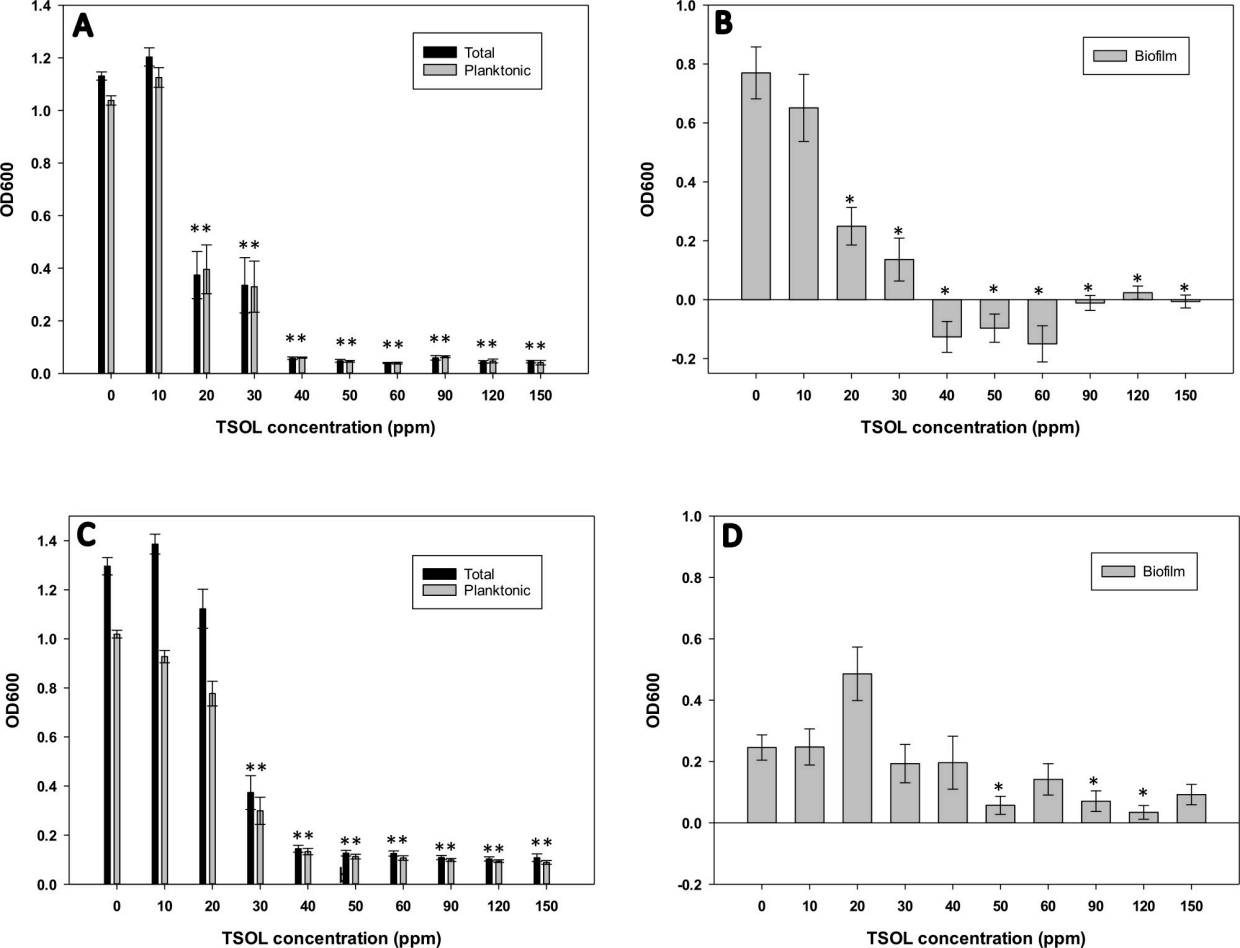
Inhibition of growth and biofilm formation by TSOL. *Xanthomonas citri* subsp. *citri* (Xcc) (A, B) and *Liberibacter crescens* (Lcr) (C, D) batch cultures in 24-well plates were treated with a TSOL concentration gradient ranging from 0–150 ppm. Total and planktonic growth (A, C) and biofilm formation (B, D) were measured by spectrophotometry (see materials and methods). Values on each graph represent means from 3 independent experiments (n = 18, with n = 6 for each treatment at each experimental repetition), and the error bars show standard error (SE) of the mean. Asterisks (*) denote statistically significant difference (*P* < 0.05) of the treatment compared to untreated control.

Xcc grows well and forms robust biofilm in SB medium compared to Nutrient Broth (NB) in MC (data not shown). Therefore, we selected SB medium to test growth and biofilm formation in batch cultures and under flow conditions in MC. The minimum inhibitory concentration (MIC) was defined as the lowest concentration of TSOL resulting in no growth of Xcc after 3 days [47, 52]. Total and planktonic growth of Xcc was significantly lower (*P* < 0.001) compared to the untreated control when Xcc was treated with TSOL concentrations of 20 ppm or higher (Fig 1A). Treatment of TSOL at concentrations of 40 ppm or higher completely inhibited the growth of Xcc, therefore 40 ppm was considered as the MIC of TSOL for Xcc. Biofilm formation of Xcc was significantly reduced using 20 ppm (*P* = 0.019) or higher (*P* < 0.001) concentrations of TSOL (Fig 1B).

In the case of Lcr total and planktonic growth was significantly lower (*P* < 0.001) compared to the untreated control when treated with 30 ppm or higher concentrations of TSOL (Fig 1C). Treatment of TSOL at a concentration of 40 ppm or higher inhibited the growth of Lcr (Fig 1C), therefore 40 ppm was considered as the MIC of TSOL for Lcr. Biofilm formation of Lcr was low in BM7 medium and the effects of biofilm formation in response to TSOL was highly variable (Fig 1D). Nevertheless inhibition of biofilm formation was observed at TSOL concentrations higher than 30ppm.

### Inhibition of growth and biofilm formation by ZnO

**Fig 2:**
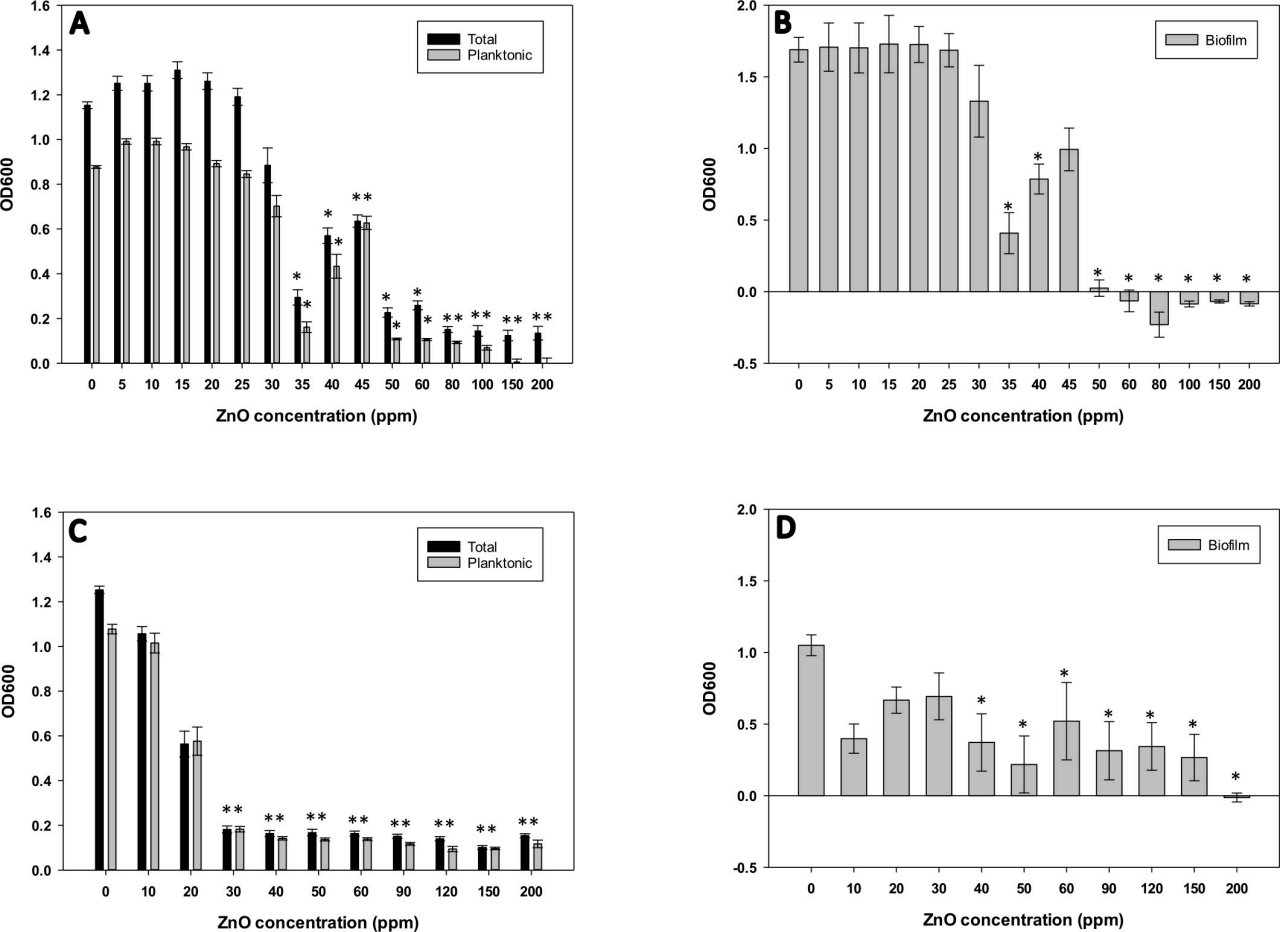
Inhibition of growth and biofilm formation by ZnO. *Xanthomonas citri* subsp. *citri* (Xcc) (A, B) and *Liberibacter crescens* (Lcr) (C, D) batch cultures in 24-well plates were treated with a ZnO concentration gradient ranging from 0–200 ppm. Total and planktonic growth (A, C) and biofilm formation (B, D) were measured by spectrophotometry (see materials and methods). Values on each graph represent means from 3 independent experiments (n = 18, with n = 6 for each treatment at each experimental repetition), and the error bars show standard error (SE) of the mean. Asterisks (*) denote statistically significant differences (*P* < 0.05) of the treatment compared to untreated control.

Antimicrobial properties of ZnO were tested on Xcc and Lcr (Fig 2) to compare its effectiveness compared to TSOL on Xcc and Lcr. Total growth and planktonic growth of Xcc was significantly lower (*P* < 0.001) compared to the untreated control when treated with 35 ppm ZnO (Fig 2A). However, there was a slight increase in both total growth and planktonic growth of Xcc when treated with 40 ppm and 45 ppm, respectively, but still the growth was significantly lower (total growth *P* = 0.001, *P* = 0.004; and planktonic growth *P* = 0.001, *P* = 0.013 for 40 ppm and 45 ppm, respectively) compared to the untreated control. Total and planktonic growth of Xcc was significantly lower (*P* < 0.001) for all the treatments at ZnO concentrations of 50 ppm or higher. However, ZnO was not bacteriostatic to Xcc in the range of ZnO concentrations tested (< 200 ppm) (Table 1). Therefore, the MIC of ZnO was considered to be greater than 200 ppm for Xcc. Biofilm formation of Xcc showed a similar pattern to that of total and planktonic growth. Xcc biofilm formation was inhibited at ZnO concentrations of 50 ppm or higher (Fig 2B).

**Table 1:** Culturability of Xcc and Lcr treated with TSOL or ZnO.

	Culturability of Xcc		Culturability of Lcr	
**Treatment (ppm)**	TSOL	ZnO	TSOL	ZnO
**0**	+	+	+	+
**10**	+	+	+	+
**20**	+	+	+	-
**30**	+	+	+	-
**40**	+	+	+	-
**50**	+	+	+	-
**60**	+	+	+	-
**90**	+	+	+	-
**120**	+	+	+	-
**150**	-	+	+	-
**200**	-	+	-	-

(+) colonies present; (-) colonies absent.

Total and planktonic growth of Lcr was significantly lower (*P* < 0.001) compared to the untreated control when Lcr was treated with 30 ppm ZnO (Fig 2C). There was no increase of growth in Lcr treated with ZnO concentrations of 30 ppm or higher. Therefore the MIC of ZnO on Lcr was established at 30 ppm. Biofilm formation of Lcr was minimal in BM7 medium and the effects of biofilm formation in response to ZnO were highly variable as observed with TSOL treatment (Fig 2D).

### Effect on cell viability of TSOL and ZnO

**Fig 3:**
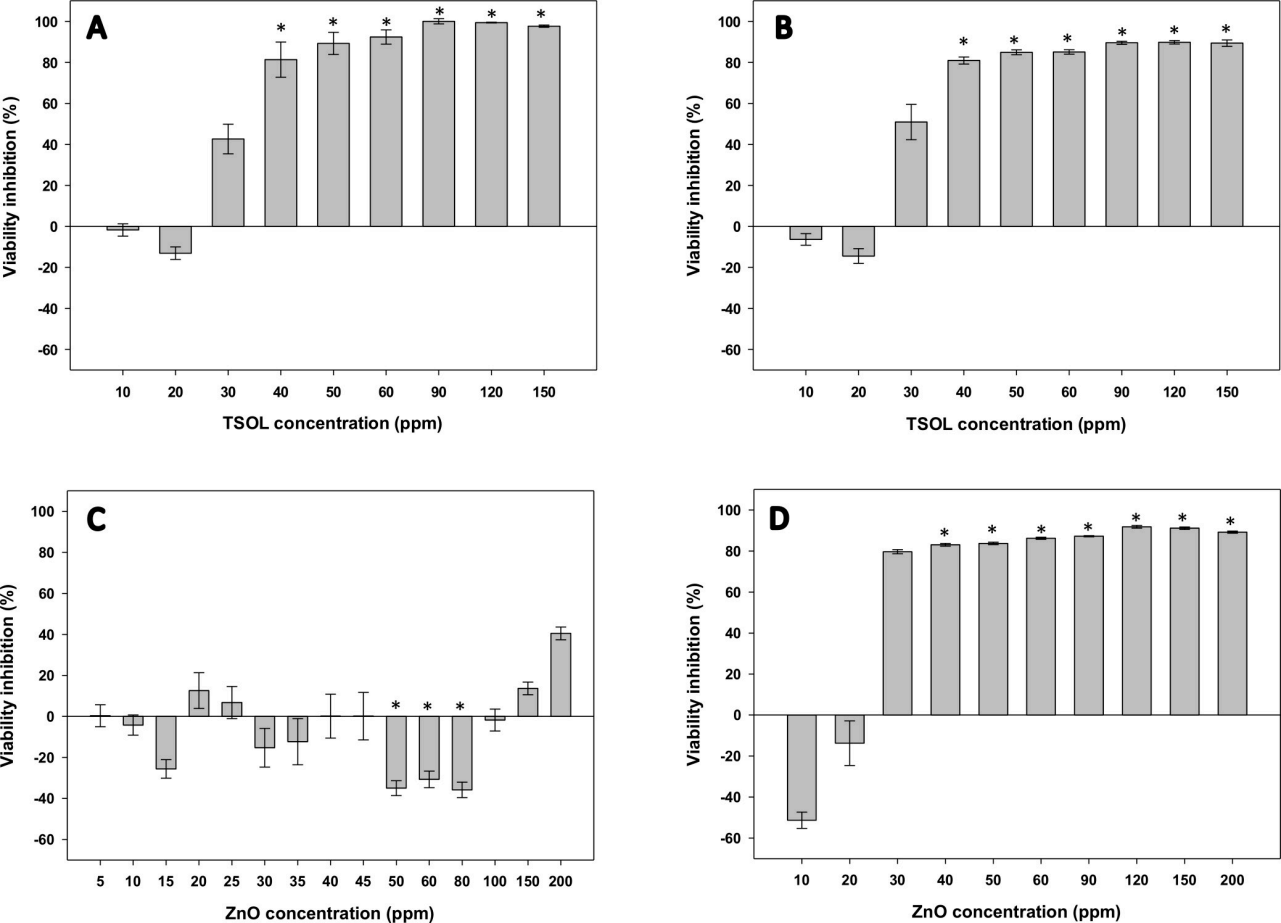
Effects of TSOL and ZnO treatments on cell viability. *Xanthomonas citri* subsp. *citri* (Xcc) (A, C) and *Liberibacter crescens* (Lcr) (B, D) batch cultures in 24-well plates were treated with a TSOL (A and B) or ZnO (C and D) concentration gradient ranging from 0–200 ppm and percentage inhibition of viability was measured using alamarBlue assay. Values on each graph represent means from 3 independent experiments (n = 18, with n = 6 for each treatment at each experimental repetition), and the error bars show standard error (SE) of the mean. Asterisks (*) denotes statistically significant differences (*P* < 0.05) of the treatment compared to untreated control.

The percentage reduction in cell viability of Xcc and Lcr after TSOL treatment was measured by alamarBlue assay (Fig 3) [53, 54]. Both Xcc and Lcr treated with TSOL showed 80% or higher reduction in cell viability at TSOL concentrations of 40 ppm or higher (Fig 3A and 3B). Conversely, reduction in Xcc viability by ZnO was much less severe. The highest inhibition of viability (40%), was observed with 200 ppm ZnO concentration (Fig 3C). Reduction in Lcr viability by ZnO treatment was much higher compared to TSOL as Lcr treated with ZnO showed 90% or higher inhibition of viability at ZnO concentrations of 30 ppm or higher (Fig 3D).

### Effects of TSOL and ZnO on culturability of bacteria

TSOL inhibited the growth (as measured by OD_600_, see above) of Xcc at TSOL concentrations of 40 ppm or higher. However, when TSOL-treated (10–120 ppm) Xcc cells were plated on SBA, colonies were observed 3–6 days after plating (Table 1). Therefore, TSOL was considered bacteriostatic to Xcc from 40 ppm up to 120 ppm [55]. However, no Xcc colonies were observed when Xcc was treated with 150 ppm of TSOL followed by plating on SBA. Therefore, TSOL was considered bactericidal to Xcc at 150 ppm [55]. When Xcc was treated with ZnO and plated on SBA plates, Xcc colonies were observed after 3–6 days even with 200 ppm ZnO treatment (Table 1). Therefore no bactericidal effect of ZnO on Xcc was observed in the concentration range tested (< 200 ppm), although a bacteriostatic growth inhibition was observed at ~35 ppm (see above).

For the case of Lcr, colonies were observed on BM7 agar plates when Lcr was treated with up to 150 ppm TSOL followed by plating. Therefore, TSOL is bacteriostatic to Lcr between 30 and 150 ppm. There were no Lcr colonies on BM7 agar plates when Lcr was treated with 200 ppm TSOL followed by plating. Therefore, TSOL was considered bactericidal to Lcr at TSOL concentrations of 200 ppm or higher (Table 1). There were no Lcr colonies on BM7 agar plates when Lcr was treated with 20 ppm or higher ZnO concentrations followed by plating. Therefore, ZnO is bactericidal to Lcr at 20 ppm or higher concentrations (Table 1).

### TSOL activity under flow conditions

**Fig 4:**
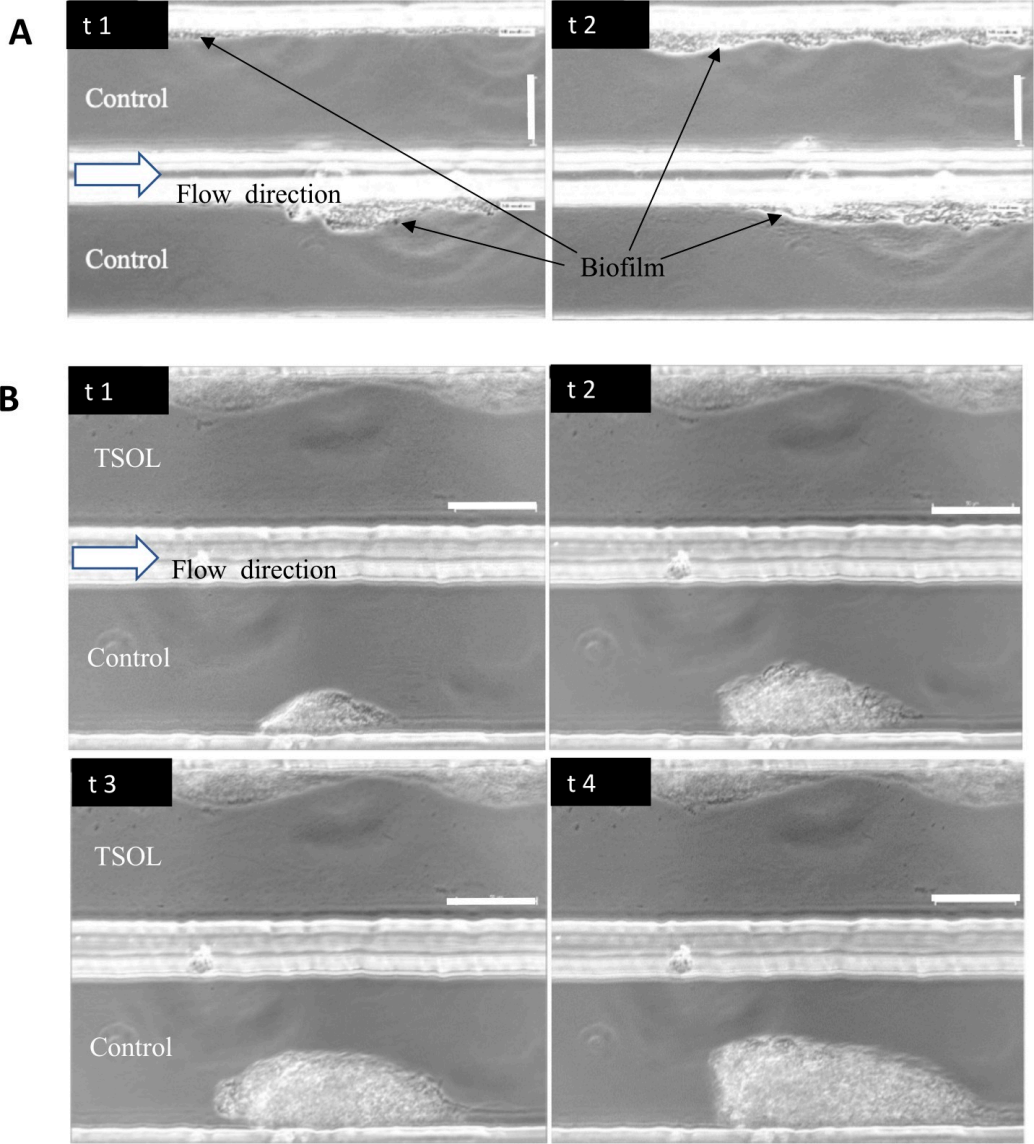
Control of biofilm growth of Xcc by TSOL. *Xanthomonas citri* subsp. *citri* (Xcc) cells were introduced to the upper and lower channels of microfluidic chambers (MC) containing SB medium (control) under constant flow of 0.05 μL/min. Sixty ppm TSOL in SB medium was introduced to the lower channel after biofilm was formed in both upper and lower channels. Growth of biofilm in treated and non-treated channels was observed by time-lapse video imaging microscopy. A) t1 = 0h, t2 = 4h. B) t1 = 0 h, t2 = 3–4 h, t3 = 4–7 h, t4 = 7–8 h. Scale bar = 50 μm. See also S1 and S2 File.

**Fig 5:**
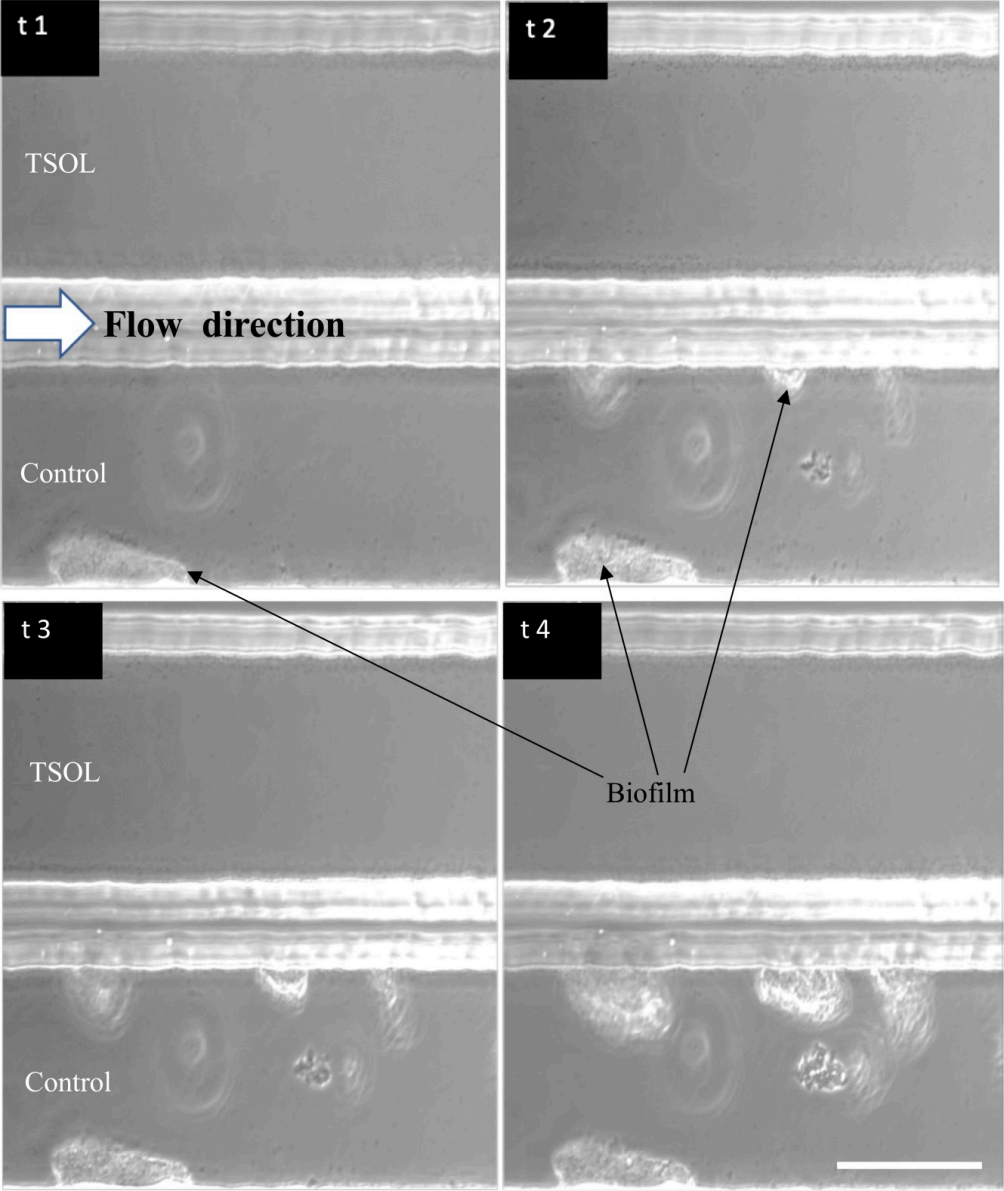
Control of biofilm growth of Lcr by TSOL. *Liberibacter crescens* (Lcr) cells were introduced to the upper and lower channels of microfluidic chambers (MC) containing bBM7+0.75mβc medium under constant flow of 0.05 μL/min. Sixty ppm TSOL in bBM7+0.75mβc medium was introduced to the lower channel after initial cell attachment in both upper and lower channels. Growth of biofilm in treated and non-treated channels was observed by time-lapse video imaging microscopy. t1 = 0 h, t2 = 30 h, t3 = 72 h, t4 = 110 h. Scale bar = 80 μm.

*C*Las is a phloem-restricted pathogen [2]. Therefore, *in vitro* evaluation of any antimicrobial compound to control *C*Las has to be tested in a comparable system to plant vascular tissues. Evaluation of TSOL on Xcc batch cultures (Fig 1B) and in MC (S1 Fig) showed that TSOL prevented initial biofilm formation of Xcc at TSOL concentrations of 40 ppm or higher in batch cultures and 60 ppm TSOL concentration in MC. Biofilms exhibit elevated antimicrobial tolerance as a result of their structural and physiological adaptations [56, 57]. Therefore, we evaluated effectiveness of TSOL to stop further growth of Xcc (Fig 4) and Lcr (Fig 5) biofilms after the initial biofilm formation using concentrations slightly higher than the determined MIC. Xcc forms biofilm in MC channels when SB medium is flowing through both MC channels at a rate of 0.05 μl/min (Fig 4A). The biofilm mass increased with time as Xcc cells divide in the biofilm. Further growth of biofilm was inhibited in the MC channels treated with SB medium containing 60 ppm TSOL at a flow rate of 0.05 μl/min, although biofilm was not disrupted. On the contrary a steady growth of Xcc biofilm was observed in the MC control channel containing only SB medium (Fig 4B).

Effects of TSOL on biofilm formation of Lcr were analyzed as described above. Lcr grows very slowly compared to Xcc in batch cultures. Furthermore, the biofilm formation and attachment of cells to the MC walls was very low in Lcr compared to that of Xcc. Therefore, Lcr was grown in bBM7+0.75mβc medium in which Lcr shows more cell attachment to MC and forms robust biofilm [49]. Lcr formed biofilm in MC channels when bBM7+0.75mβc medium flows through both MC channels (Fig 5). Lcr cells attached to the MC channel containing only the bBM7+0.75mβc medium showed an increase in the amount of biofilm over time, whereas Lcr cells attached to the MC channel treated with 60 ppm TSOL in bBM7+0.75mβc medium did not divide or show any increase in biofilm (Fig 5).

### Plant uptake and movement of TSOL

**Fig 6:**
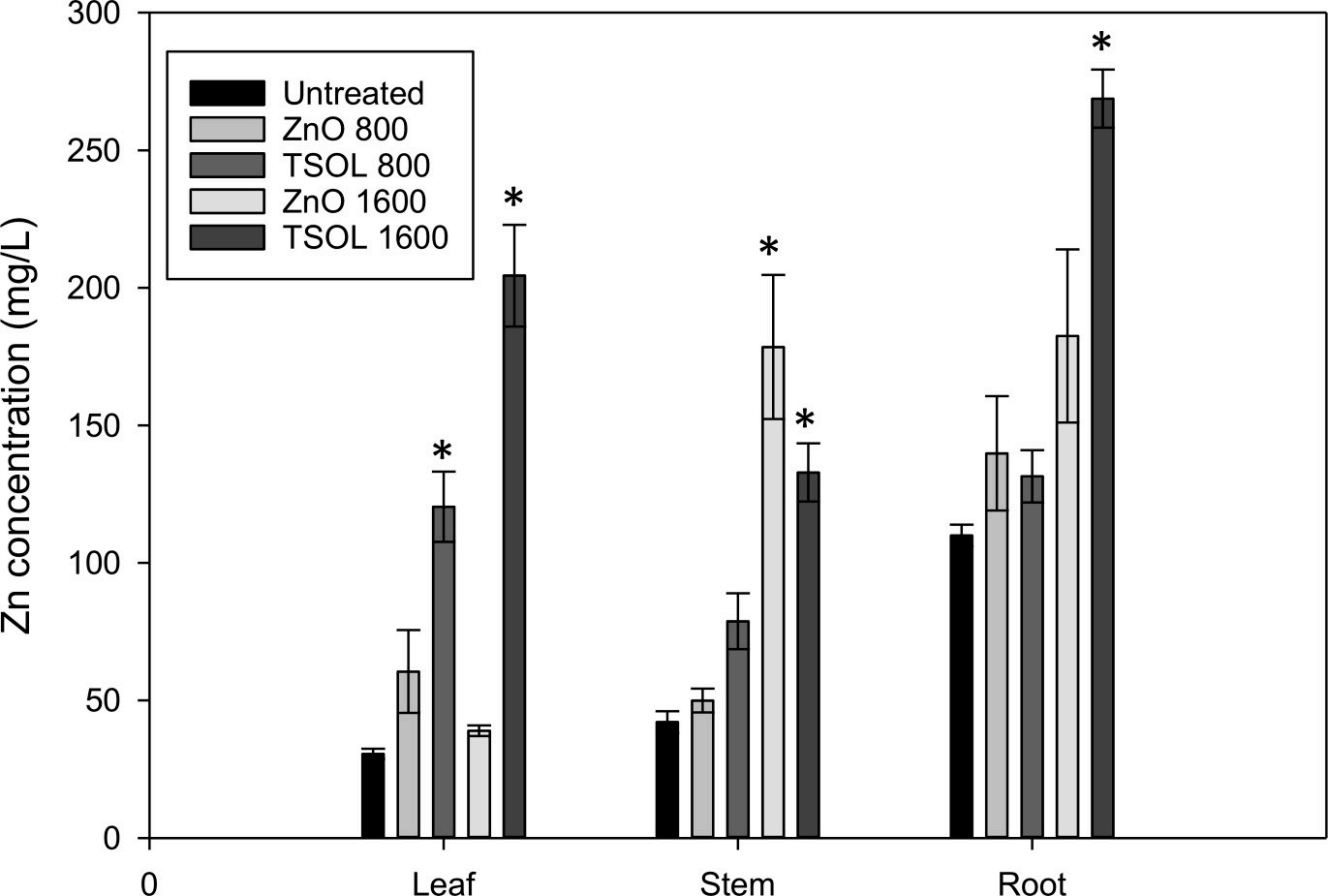
Plant uptake of ZnO and TSOL through leaf spraying. *Citrus reshni* seedlings were sprayed with 800 μg Zn/mL and 1600 μg Zn/mL of ZnO and TSOL. Zn content in leaves, stem and roots were measured two days after treatment application by Atomic Absorption Spectrometry. Values on each graph represent means from 2 independent experiments (n = 8, with n = 4 for each treatment at each experimental repetition), and the error bars show standard error (SE) of the mean. Asterisks (*) denote statistically significant differences (*P* < 0.05) of treatments compared to control.

TSOL should have better plant uptake by leaves and systemic movement in the phloem to control systemic plant pathogens such as *C*Las. Therefore, we evaluated Zn uptake and movement in citrus plants by spraying leaves with TSOL and bulk ZnO. According to our results, 800 μg Zn/mL and 1600 μg Zn/mL treatments of TSOL had significantly higher uptake in leaves (*P* = 0.002 and *P* < 0.001, respectively) compared to the same concentrations of ZnO and also the untreated control (Fig 6). The 1600 μg Zn/mL TSOL treatment had significantly higher (*P* = 0.05) Zn uptake by leaves than 800 μg Zn/mL TSOL treatment. Significantly higher Zn content was found in stems of the citrus plants treated with 1600 μg Zn/mL TSOL and ZnO treatments (*P* < 0.001). However, there was no significant difference in Zn content between the stems treated with 1600 μg Zn/mL of TSOL and ZnO (Fig 6). There was significantly higher (*P* < 0.001) Zn content in the roots of citrus plants treated with 1600 μg Zn/mL TSOL (Fig 6). According to our results TSOL has significantly higher leaf uptake and movement to roots compared to that of ZnO.

## Discussion

Currently, no viable economical treatment exists to reduce *C*Las populations in HLB-affected trees or to reverse the decline of HLB-affected trees [58]. Thermal therapy of HLB-affected plants has been tested previously, but lack of treatment effects in the root system where *C*Las is found is a problem [58]. Elimination of *C*Las from the entire citrus tree through thermal therapy may be impossible without a chemical treatment [58]. Trunk injection of plant defense activators and antibiotics has been tested and showed reduction in *C*Las populations and HLB symptom expression [59–61]. However, trunk injection is labor intensive and is not an economically sustainable strategy. There are several effective ACP repellants including sulfur volatiles and essential oils that have been shown to be successful in the field, but they are not available to citrus growers since there are no commercially available application methods for these compounds [62–64]. The Kaolin clay-based ACP repellant SurroundWP has shown good efficacy in altering ACP feeding behavior and is commercially available [65, 66]. However, without an antimicrobial treatment against *C*Las, ACP repellant alone will not be enough to control the spread of HLB. Therefore, a comprehensive HLB management solution that is industrially viable, affordable to growers and sustainable and also has minimal negative impact on the environment and human health is required.

MS3T technology has been developed by combining several products customized to deliver comprehensive HLB and citrus canker control [27]. MS3T is made of chemicals which are either naturally occurring, “Generally Recognized as Safe” (GRAS) by the USA Food and Drug Administration (FDA) or the USA Environmental Protection Agency (EPA) approved for “Food Use Only” [67, 68]. The current MS3T formulation consists of Kaolin clay, a quaternary ammonium compound (Fixed-Quat) and TSOL. A naturally-occurring aluminosilicate, Kaolin clay is an ACP repellent and is used as a delivery system for TSOL [27, 69, 70]. Fixed-Quat is an antimicrobial compound and was designed to control surface and sub-surface restricted pathogens such as Xcc. TSOL is a water soluble Zn-chelate [27] and is designed to be a plant tissue permeable systemic bactericide [27]. As we describe here, TSOL can be absorbed through leaves and transported to stems and root systems most likely through phloem flow. Therefore, it is expected to be effective against systemic plant pathogens such as *C*Las.

There are many reports of antimicrobial properties of ZnO [31–38], the most common Zn-based chemical commercially available that was used here for comparison. Antimicrobial properties of ZnO are based on the catalysis of formation of ROS from water and oxygen and the release of Zn ions [35, 39, 40, 71–73]. These properties are directly dependent of the particle size of the ZnO. Bulk ZnO is not easily taken up by plants and has limited systemic movement in plants as a result of its bigger particle size. Therefore, bulk ZnO as an antimicrobial compound is ineffective in controlling systemic plant pathogens. Nano-ZnO formulations such as Zinkicide has better antimicrobial activity as well as proposed systemic movement in plants [20]. However, Zinkicide is not commercially available to citrus farmers at this time. Therefore, Zn-chelates such as TSOL could offer antimicrobial activity similar to Nano-ZnO formulations with better plant uptake and could be fast-tracked for EPA registration.

According to studies of antimicrobial activity of ligands and its complexes, metal chelates have higher antimicrobial activity than their free ligand [74, 75]. Polarity of a metal ion is reduced as a result of overlapping ligand orbital and partial sharing of the positive charge with donor groups [74]. Therefore, metal chelates are more lipophilic than free ligands. Higher liposolubility of metal chelates could be partly responsible for higher antimicrobial activity of metal chelates compared to that of free ligands. Therefore, metal chelates can penetrate lipid membranes and block metal binding sites on the enzymes of bacteria [74]. We observed a higher antimicrobial activity in TSOL compared to bulk ZnO against Xcc. However, that was not the case against Lcr. Since, TSOL is designed to be a systemic antimicrobial compound, TSOL is more effective than bulk ZnO against systemic pathogens such as *C*Las by reaching Zn concentrations higher than MIC in planta. [27]. Our results from plant uptake and movement of Zn in citrus plants treated with TSOL and ZnO show significantly higher amounts of TSOL taken up by citrus leaves and this leads to significantly higher Zn concentrations in the roots of citrus plants compared to those of ZnO treated plants. Therefore, our plant Zn uptake study shows that even though ZnO has slightly better antimicrobial activity against Lcr, its limited uptake and movement in plants may hinder its effectiveness as a systemic bactericide. Uptake of TSOL by the leaves, basipetal movement and the resulting high TSOL concentrations in the roots within two days after treatment application all support the movement of TSOL through the phloem. *C*Las infects phloem and moves from infected leaves to roots and back to young leaves through the phloem [76]. Therefore, higher TSOL concentrations in the leaves, stems and roots should help to lower *C*Las populations in planta.

The MIC of an antimicrobial agent is the lowest concentration of the antimicrobial compound that can kill (bactericidal activity) or inhibit (bacteriostatic activity) the growth of bacteria [52]. Therefore, the MIC values do not indicate whether a particular antimicrobial compound is bacteriostatic or bactericidal. Plating of treated bacteria in respective growth medium helps to determine whether the antimicrobial compound is bactericidal or bacteriostatic. However, some pathogenic and non-pathogenic bacteria can enter a viable but non-culturable (VBNC) state when they are exposed to stress [77]. Bacteria in a VBNC state fail to grow on the routine growth media but they are alive and are capable of renewed metabolic activity [78]. Processes that are normally bactericidal to bacteria could also lead to a VBNC state in bacteria [79]. Del Campo et al. (2009) reported that Xcc can enter a VBNC state after copper sulfate treatment for a short time [80]. Several methods have been described to determine viable counts of bacteria in a VBNC state [81–83]. We evaluated the viability of Xcc and Lcr by the alamarBlue assay (Fig 3) [84–86]. However, we did not observe Xcc or Lcr entering a VBNC state after TSOL or ZnO treatments according to an oxidation-reduction indicator. According to Del Campo et al. (2009) the VBNC state in Xcc was induced by a 10 min treatment of 135 μM CuSO_4_ but a 30 min treatment did not induce a VBNC state in Xcc [80]. VBNC state in Lcr after a treatment with an antimicrobial compound has not been previously recorded. Since MS3T is designed to form a layer on citrus leaves and fruits, and to release TSOL over time [27], the probability of Xcc exposure to a high dose of TSOL is very low in field conditions. Therefore, our results suggest it is unlikely that Xcc or Lcr will enter the VBNC state under field conditions.

Culturability of Xcc after TSOL treatment indicates that TSOL is bacteriostatic to Xcc at concentrations between 40–120 ppm, and bactericidal at 150 ppm or higher. ZnO is not bactericidal to Xcc up to 200 ppm. According to culturability data after TSOL treatment, TSOL is bacteriostatic to Lcr from 40–150 ppm and bactericidal to Lcr at 200 ppm. ZnO is bactericidal to Lcr at 20 ppm or higher ZnO concentrations. Previous studies on antimicrobial activity of ZnO have shown that ZnO is more effective against Gram-positive bacteria than Gram-negative bacteria [33, 35, 38, 87]. The differences in antimicrobial activity of ZnO within Gram-negative bacteria may result from antioxidant cellular content and resistance to oxidative stress in different bacteria species [73]. We observed such differences in antimicrobial activity of ZnO against Xcc and Lcr. However, there was no such difference in antimicrobial activity of TSOL against Xcc and Lcr. Graham et al (2016) reported that a ZnO nano formulation with 62.5 ppm MIC against *Xanthomonas alfalfae* was effective in reducing citrus canker lesion development in citrus leaves [20]. We have determined the MIC of TSOL against Xcc to be 40 ppm. Therefore, we can expect similar efficacy of TSOL against citrus canker.

Many bacteria in the natural environment form biofilm when they are associated with solid surfaces [56, 57, 88, 89]. Bacteria cells in biofilm are physiologically different from those of planktonic cells [90, 91]. Biofilms have higher tolerance to antimicrobial compounds as a result of their distinct physiology and biofilm structure [57]. Plant pathogenic bacteria form various multicellular aggregations on plant surfaces. A few other plant pathogens form multicellular aggregates in phloem and xylem and they are generally dependent on insect vectors or wounding for infection [92–95]. *C*Las form extensive biofilm in ACP alimentary canals whereas biofilm formation of *C*Las in planta has not been reported [76, 96, 97]. We evaluated effectiveness of TSOL to control biofilm formation of Xcc and Lcr in MC under similar conditions found in vascular tissues of plants. Studies of biofilm formation of vascular plant pathogens such as *Xylella fastidiosa* in MC have provided insights of pathogenicity and possible treatments options [48, 98, 99]. TSOL was effective against at controlling growth and biofilm formation of Xcc and Lcr in batch cultures as well as in MC. Control of biofilm formation of Xcc required slightly higher TSOL concentrations (60 ppm) than MIC (40 ppm) for Xcc in batch cultures. A 60 ppm TSOL concentration prevented the growth of already formed Xcc and Lcr biofilm. However, it did not disrupt already formed Xcc and Lcr biofilms in MC. MIC values, investigation of viability with plating and alamarBlue assays, experiments under flow conditions (MC), TSOL uptake by citrus leaves and systemic movement of TSOL show that it has potential as an antimicrobial treatment to control growth and biofilm formation of plant surface pathogens such as Xcc and systemic plant pathogens such as *C*Las. We could not investigate effectiveness of TSOL against *C*Las in vitro since *C*Las is not culturable. Therefore, ongoing field efficacy testing of TSOL on *C*Las affected plants will provide a better understanding of the effectiveness of TSOL against *C*Las. We did not observe any signs of phytotoxicity in citrus leaves after TSOL treatment (S2 Fig). Hippler et al (2015) have studied uptake and distribution of Zn by citrus plants after Zn treatment and found that citrus fruits are not sinks of zinc [100]. Application of the ^68^Zn isotope showed significant increases of Zn levels in roots, trunks, branches, leaves and flowers. However, there was no significant increase in Zn levels in fruits [100]. Furthermore, oral LD50 of Zn(NO_3_)_2_ is 1190 mg/kg which is two-fold higher than that of commonly used antimicrobial compound CuSO_4_ [101, 102]. Efficacy of TSOL is currently being tested in the field to control HLB and citrus canker.

## Supporting information

S1 Fig Evaluation of effects of TSOL on biofilm formation of Xcc in microfluidic chambers. *Xanthomonas citri* subsp. *citri* (Xcc) was introduced to upper and lower microfluidic chamber (MC) channels while SB medium was flowing through the upper channel with the flow rate of 0.05 μl/min and the lower channel was treated with SB medium containing 60 ppm with a flow rate of 0.05 μl/min. t1 = 0 h, t2 = 2–3 h, t3 = 5–6 h, t4 = 8–9 h, t5 = 10 h. Scale bar = 80 μm.
(TIFF)

S2 Fig Evaluation of phytotoxicity of TSOL, ZnO and copper sulfate after foliar spraying. **a)** DI water. **(b)** TSOL at 800 μg Zn /mL, **(c)** TSOL at 1,600 μg Zn /mL, **(d)** ZnO 400 at 800 μg Zn/mL, **(e)** ZnO 400 at 1,600 μg Zn/mL, **(f)** Copper sulfate at 1,600 μg Cu/mL. No injury on plants was observed for all materials at tested concentrations. Images were taken 72 hours after leaf spraying.
(TIFF)

S1 File Growth of *Xanthomonas citri* subsp. *citri* in microfluidic chambers. *Xanthomonas citri* subsp. *citri* (Xcc) cells were introduced in both channels of microfluidic chambers (MC) containing SB medium (control) under constant flow of 0.05 μL/min. Movie shows growth and biofilm formation observed by time-lapse video imaging microscopy. Scale bar = 50 μm. Movie corresponds to images shown in Fig 4A.
(MP4)

S2 File Effect of TSOL on *Xanthomonas citri* subsp. *citri* growth and biofilm formation in microfluidic chambers. *Xanthomonas citri* subsp. *citri* (Xcc) cells were introduced to the upper and lower channels of microfluidic chambers (MC) containing SB medium under constant flow of 0.05 μL/min. 60 ppm TSOL in SB medium was introduced to the lower channel after biofilm was formed in both upper and lower channels. Growth of biofilm in treated and non-treated channels was observed by time-lapse video imaging microscopy. Scale bar = 50 μm. Movie corresponds to images shown in Fig 4B.
(MP4)
