# Supplementary figures and images for: A novel Zn chelate (TSOL) that moves systemically in citrus plants inhibits growth and biofilm formation of bacterial pathogens
Source: PLoS One. 2019 Jun 24;14(6):e0218900. doi: 10.1371/journal.pone.0218900 (PMC6590827; doi:10.1371/journal.pone.0218900)

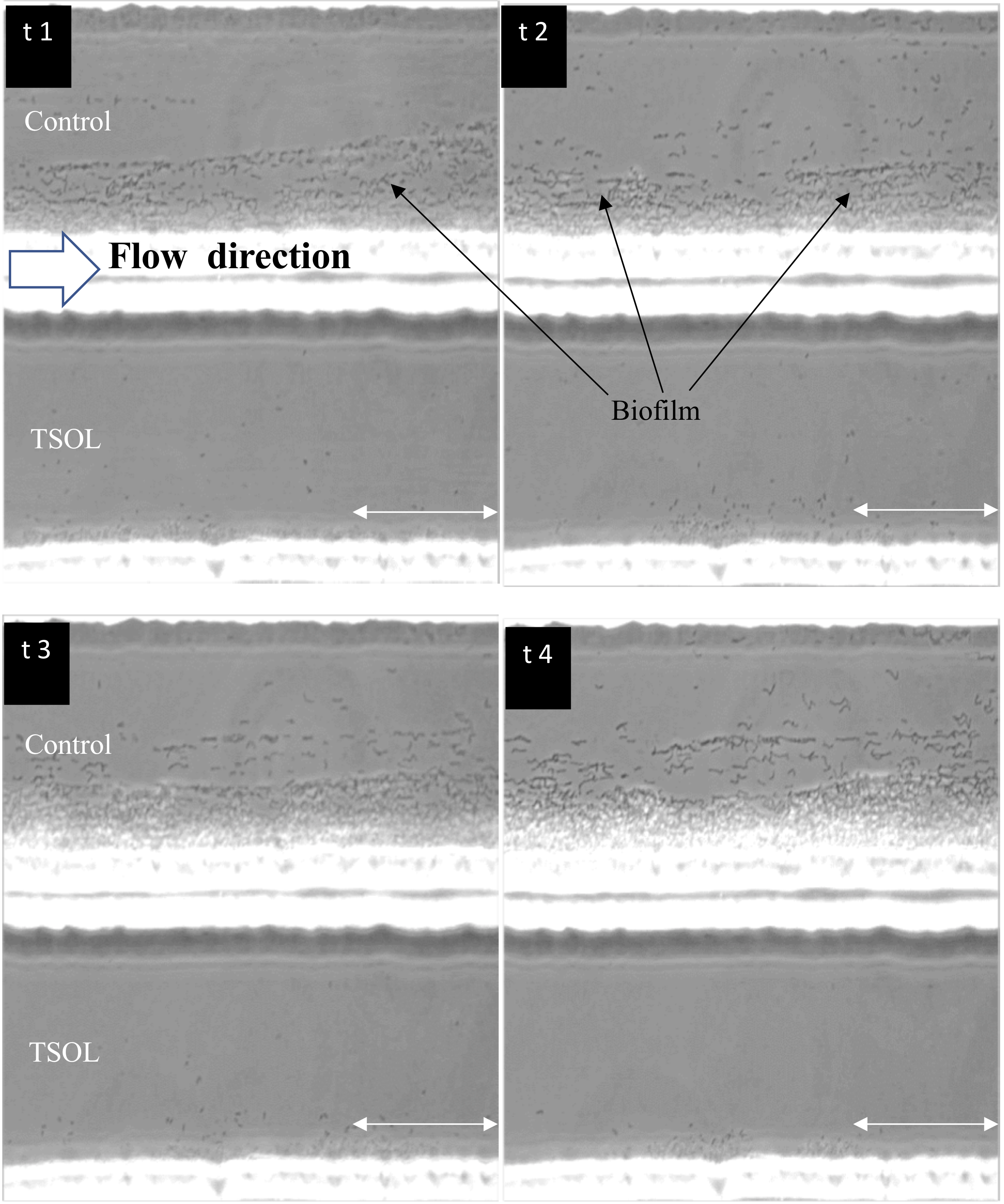

Supplement: S1 Fig — Xanthomonas citri subsp. citri (Xcc) was introduced to upper and lower microfluidic chamber (MC) channels while SB medium was flowing through the upper channel with the flow rate of 0.05 μl/min and the lower channel was treated with SB medium containing 60 ppm with a flow rate of 0.05 μl/min. t1 = 0 h, t2 = 2–3 h, t3 = 5–6 h, t4 = 8–9 h, t5 = 10 h. Scale bar = 80 μm. (TIFF) [file pone.0218900.s001.tiff]

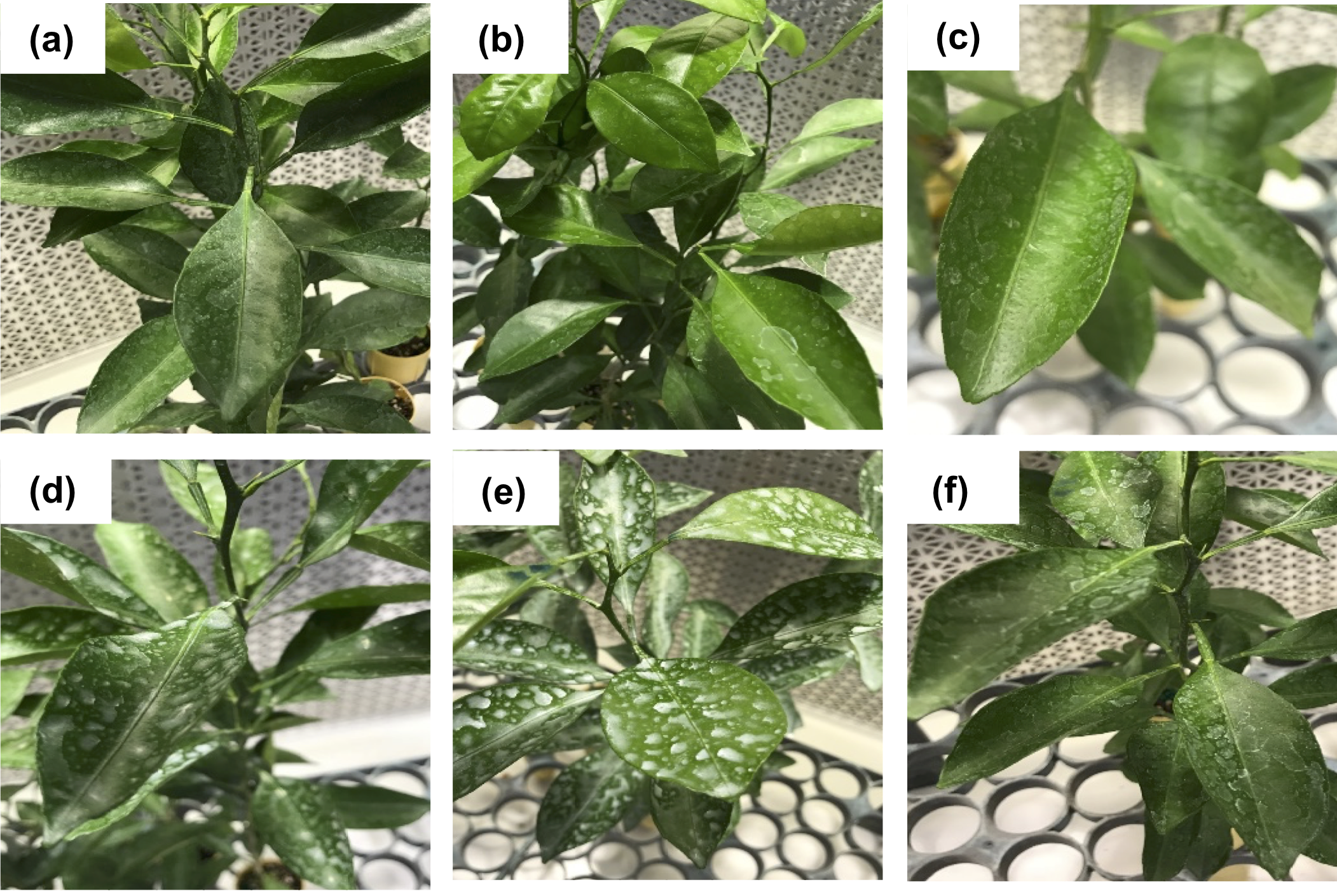

Supplement: S2 Fig — a) DI water. (b) TSOL at 800 μg Zn /mL, (c) TSOL at 1,600 μg Zn /mL, (d) ZnO 400 at 800 μg Zn/mL, (e) ZnO 400 at 1,600 μg Zn/mL, (f) Copper sulfate at 1,600 μg Cu/mL. No injury on plants was observed for all materials at tested concentrations. Images were taken 72 hours after leaf spraying. (TIFF) [file pone.0218900.s002.tiff]
